# Subphenotyping prone position responders with machine learning

**DOI:** 10.1186/s13054-025-05340-8

**Published:** 2025-03-14

**Authors:** Maxime Fosset, Dario von Wedel, Simone Redaelli, Daniel Talmor, Nicolas Molinari, Julie Josse, Elias N. Baedorf-Kassis, Maximilian S. Schaefer, Boris Jung

**Affiliations:** 1 Department of Anesthesia, Critical Care and Pain Medicine, Beth Israel Deaconess Medical Center, Harvard Medical School, Boston, MA USA; 2 Center for Anesthesia Research Excellence (CARE), Beth Israel Deaconess Medical Center, Harvard Medical School, Boston, MA USA; 3 Medical Intensive Care Unit and PhyMedExp, Lapeyronie Montpellier University Hospital, Lapeyronie Teaching Hospital, University Montpellier, 1; 371 Avenue Du Doyen Gaston Giraud, 34090 Montpellier, CEDEX 5, France; 4 Desbrest Institute of Epidemiology and Public Health, University of Montpellier, INRIA, Montpellier, France; 5 Institute of Medical Informatics, Charité-Universitätsmedizin Berlin, Berlin, Germany; 6 School of Medicine and Surgery, University of Milano-Bicocca, Milan, Italy; 7 Department of Anesthesiology, Perioperative and Pain Medicine, Lahey Hospital and Medical Center, Burlington, MA USA; 8 Department of Pulmonary, Critical Care and Sleep Medicine, Beth Israel Deaconess Medical Center, Harvard Medical School, Boston, MA USA; 9 Department of Anesthesiology, Duesseldorf University Hospital, Duesseldorf, Germany

**Keywords:** Prone Position, ARDS, Clustering, Phenotypes, Machine Learning, Precision Medicine

## Abstract

**Background:**

Acute respiratory distress syndrome (ARDS) is a heterogeneous condition with varying response to prone positioning. We aimed to identify subphenotypes of ARDS patients undergoing prone positioning using machine learning and assess their association with mortality and response to prone positioning.

**Methods:**

In this retrospective observational study, we enrolled 353 mechanically ventilated ARDS patients who underwent at least one prone positioning cycle. Unsupervised machine learning was used to identify subphenotypes based on respiratory mechanics, oxygenation parameters, and demographic variables collected in supine position. The primary outcome was 28-day mortality. Secondary outcomes included response to prone positioning in terms of respiratory system compliance, driving pressure, PaO_2_/FiO_2_ ratio, ventilatory ratio, and mechanical power.

**Results:**

Three distinct subphenotypes were identified. Cluster 1 (22.9% of whole cohort) had a higher PaO_2_/FiO_2_ ratio and lower Positive End-Expiratory Pressure (PEEP). Cluster 2 (51.3%) had a higher proportion of COVID-19 patients, lower driving pressure, higher PEEP, and higher respiratory system compliance. Cluster 3 (25.8%) had a lower pH, higher PaCO_2_, and higher ventilatory ratio. Mortality differed significantly across clusters (p = 0.03), with Cluster 3 having the highest mortality (56%). There were no significant differences in the proportions of responders to prone positioning for any of the studied parameters. Transpulmonary pressure measurements in a subcohort did not improve subphenotype characterization.

**Conclusions:**

Distinct ARDS subphenotypes with varying mortality were identified in patients undergoing prone positioning; however, predicting which patients benefited from this intervention based on available data was not possible. These findings underscore the need for continued efforts in phenotyping ARDS through multimodal data to better understand the heterogeneity of this population.

**Supplementary Information:**

The online version contains supplementary material available at 10.1186/s13054-025-05340-8.

## Background

Acute respiratory distress syndrome (ARDS) is a common and severe cause of admission to the intensive care unit (ICU) with a mortality rate of up to 45% [1, 2]. ARDS can be defined as a heterogeneous phenotype of clinical, biological, and radiological signs secondary to pulmonary or extrapulmonary aggression [2, 3]. Recent guidelines have stressed the importance of identifying subphenotypes based on data-driven assessment of multimodal traits [4]. Subphenotyping is a challenging task that requires collection of clinically relevant variables related to the pathophysiology of ARDS to provide personalized care [5] or prognostic enrichment. To personalize therapeutic strategies, biological and clinical hyper-inflammatory and hypo-inflammatory subphenotypes of ARDS have been described, which may benefit from different levels of positive end-expiratory pressure (PEEP) [6, 7] or fluid strategies [8], and can be identified early [9].

Prone positioning [10] is recommended for moderate-to-severe ARDS [4] and remains one of the most useful techniques for decreasing mortality in this population [11]. Its utilization has increased greatly during the coronavirus disease 2019 (COVID-19) pandemic [12]. However, apart from findings on prone positioning being most beneficial in a subgroup with a PaO_2_/FiO_2_ ratio under 150 mmHg, little is known about potential ARDS subphenotypes that might benefit the most or could even be harmed from the technique [13, 14]. While the LIVE trial [15] showed no significant mortality difference in the intention-to-treat analysis, its per-protocol findings suggest a potential benefit of personalized proning strategies, emphasizing the importance of tailored approaches in ARDS management.

Nonetheless, the question of which subphenotype might benefit most from prone positioning subsists, as patients without improvement in gas exchange may still benefit [16]. The morphological subphenotype of ARDS, assessed through chest radiography, ultrasound, or computed tomography, fails to predict improvement in oxygenation during proning [17]. Recent machine learning methods used on electronic health record data failed to predict the success of prone positioning during COVID-19 related ARDS, defined as an improvement of at least 10% in the PaO_2_/FiO_2_ ratio, ventilatory ratio, respiratory system compliance, or mechanical power [18]. Additionally, Hannon et al. [19] highlighted the potential of machine learning in predicting mortality after prone positioning despite poor model performance in terms of specificity. Furthermore, it is unknown whether transpulmonary pressure measurements can improve identification of subphenotypes. As a key element in the personalization of mechanical ventilation, esophageal pressure monitoring, which allows for the estimation of transpulmonary pressures [20–22] and transpulmonary mechanical power [23], can improve the assessment and quantification of the risk of lung injury [24].

In this study, we aimed to identify subphenotypes of patients undergoing prone positioning based on respiratory system mechanics, transpulmonary pressure measurements, and gas exchange parameters and assess their association with mortality and response to prone position.

## Methods

### Study population and data collection

In this retrospective observational study, we enrolled all consecutive critically ill adult patients receiving invasive mechanical ventilation for at least 24 h and at least one cycle of prone positioning between 2020 and 2022 at the Beth Israel Deaconess Medical Center in Boston, Massachusetts. Prone positioning was performed in accordance with a standardized institutional protocol (E-Table 1), which detailed criteria for initiation, duration, and discontinuation based on ARDS severity. PEEP levels were adjusted based on individual patient characteristics and clinical judgment rather than a standardized protocol. Esophageal manometry was used at the discretion of the attending clinicians, without strict protocol, to guide PEEP adjustments. These adjustments were based on achieving target transpulmonary pressures, with a goal expiratory transpulmonary pressure of -2 to + 2 cmH₂O and an inspiratory transpulmonary pressure ≤ 15 cmH₂O, as per the clinical team's judgment. Deidentified data were extracted from the electronic health records of the eligible patients. Respiratory parameters during the 12 h preceding the first cycle of proning, the period of proning, and 12 h after the first cycle of prone positioning were derived and changes between these periods calculated. If several measurements for a variable in a period were available, medians were calculated after cleaning for clinically implausible values for the respective parameter [25]. Patients with missing data for critical variables necessary to calculate mechanical power specifically respiratory rate (RR), tidal volume (Vt), peak inspiratory pressure (PIP), and positive end-expiratory pressure (PEEP), were excluded from analyses. Other missing covariates were deemed to be missing at random and imputed through a single imputation algorithm using a Factorial Analysis for Mixed Data (FAMD) model. This allows for imputation, considering similarities between both individuals and relationships between variables and further dimension reduction analysis [26]. This approach was employed to preserve the sample size and minimize potential biases that might emerge from excluding patients with incomplete data. During each period, mechanical power, driving pressure, and respiratory system compliance were computed for each patient. Mechanical power was calculated from the median ventilator parameters, using the following equation: mechanical power (J/min) = 0.098 × RR × Vt × (PIP – ½ (Pplat-PEEP)) [27]. The ventilatory ratio was computed as [minute ventilation (ml/min) × PaCO_2_ (mmHg)]/(predicted body weight × 100 × 37.5) [28].

**Table 1:** Supine position characteristics of the overall population and of the three clusters

Characteristic	Overall N = 353^a^	Cluster 1 N = 81^a^	Cluster 2 N = 181^a^	Cluster 3 N = 91^a^
Age (years)	62 (52, 69)	66 (53, 75)	61 (51, 69)	61 (50, 68)
Sex (female)	141 (40%)	22 (27%)	76 (42%)	43 (47%)
COVID-19 Positive	252 (71%)	38 (47%)	153 (85%)	61 (67%)
Body Mass Index (BMI, kg/m^2^)	31 (27, 36)	27 (24, 31)	32 (29, 36)	33 (27, 42)
Elixhauser Comorbidity Score	22 (16, 31)	28 (20, 40)	20 (13, 27)	24 (17, 32)
Obstructive Lung Disease	108 (31%)	28 (35%)	52 (29%)	28 (31%)
Restrictive Lung Disease	35 (10%)	4 (5%)	9 (5%)	22 (24%)
Smoking Status	144 (41%)	36 (44%)	73 (40%)	35 (38%)
Total SOFA Score	10 (6, 12)	9 (6, 12)	10 (6, 12)	11 (7, 14)
Median Respiratory Rate (breaths/min)	26 (22, 30)	21 (18, 24)	26 (24, 29)	30 (28, 32)
Median PEEP (cmH_2_O)	12 (10, 14)	8 (5, 10)	13 (12, 15)	12 (10, 16)
Median Tidal Volume (mL)	380 (330, 440)	421 (380, 470)	380 (330, 425)	350 (300, 410)
Predicted Body Weight (kg)	60 (52, 66)	64 (55, 69)	60 (52, 66)	60 (52, 65)
Median Tidal Volume per kg PBW (mL/kg)	6.1 (5.8, 6.6)	6.4 (6, 7)	6.1 (5.9, 6.5)	6 (5.5, 6.3)
Median Plateau Pressure (cmH_2_O)	25 (22, 28)	21.5 (18, 24)	25 (23, 27)	29.5 (27, 32)
Mechanical Power (J/min)	21 (16, 25)	13 (7, 18)	21 (18, 25)	25 (21, 31)
Median PaO_2_ (mmHg)	88 (77, 101)	93 (78, 115)	86 (76, 95)	87 (78, 102)
Median FiO_2_ (%)	74 (60, 90)	60 (50, 78)	70 (60, 80)	90 (80, 100)
Median PaO_2_/FiO_2 _Ratio	126 (97, 152)	165 (127, 220)	126 (99, 142)	103 (84, 130)
Median PaCO_2_ (mmHg)	49 (43, 58)	45 (40, 51)	47 (43, 53)	61 (53, 66)
Median pH	7.33 (7.28, 7.37)	7.36 (7.32, 7.40)	7.34 (7.30, 7.38)	7.27 (7.22, 7.31)
Median Driving Pressure (cmH_2_O)	12.5 (10, 15.7)	12.5 (9, 15.9)	11.5 (10, 13.4)	16 (13.1, 20)
Median Respiratory System Compliance (mL/ cmH_2_O)	31 (23, 40)	35 (25, 47)	33 (27, 41)	21 (16, 29)
Ventilatory Ratio	2.1 (1.7, 2.6)	1.6 (1.4, 1.8)	2 (1.8, 2.4)	2.8 (2.4, 3.3)

^a^Median (IQR); n (%)

When several measurements were available, the median was computed for each patient. The SOFA score was computed using data on its primary components during the first 24 h after admission. These components included PaO_2_, FiO_2_, platelets, bilirubin, mean arterial pressure and use of vasoactive agents, creatinine, mechanical ventilation, and the Glasgow Coma Scale. ICD codes used to define obstructive and restrictive lung disease are shown in E-Table 10. **Abbreviations: SOFA:** Sepsis-related Organ Failure Assessment [40], **PEEP:** Positive End-expiratory Pressure, **PBW**: Predicted Body Weight

The transpulmonary driving pressure, lung compliance, chest wall compliance, and esophageal expiratory pressure were extracted for the subcohort of patients with available esophageal manometry measurements before, during, and after prone position, and were analyzed as part of the full cohort and as a standalone cohort.

### Subphenotyping by clustering analysis

To identify unobserved clusters of patients, based on respiratory parameters, that may have differential prognosis and response to prone positioning, we used unsupervised machine learning.

We used factorial analysis of mixed data, an extension of principal component analysis suited to mixed (quantitative and qualitative) data. This approach allows to summarize the information by reducing the dimensionality of the data while retaining important information.

The hierarchical clustering method was then used to define three clusters and was built by computing the Euclidean distance between individuals and using the Ward criterion to minimize the variance at each step of the construction of the algorithm and homogenize the clusters [29] (E-Fig. 1).

**Fig. 1:**
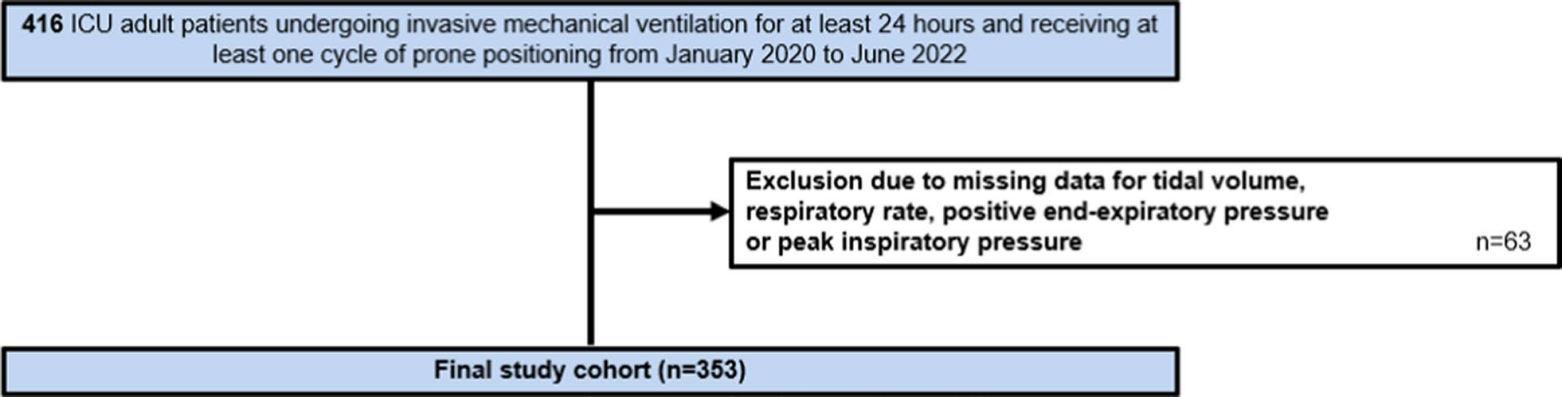
Flowchart

The clusters are described with a v-test or a Pearson’s chi-squared test when appropriate to assess the difference between the cluster and the overall population for the variables of interest.

### Association of subphenotypes with mortality and prone positioning response

The primary outcome was 28-day mortality. Secondary outcomes included response to prone positioning, defined as any improvement during prone positioning compared to the supine position, specifically a change > 0 for respiratory system compliance and PaO_2_/FiO_2_ ratio, or a change < 0 for driving pressure, ventilatory ratio, and mechanical power. [18]. Mortality was assessed through the national death registry through hospital network if death occurred outside the hospital [30].

Mortality and the proportion of prone position responders were compared between clusters using Pearson’s chi-squared tests with Holm-Bonferroni correction for multiple testing. Threshold for significance was set to 0.05 unless stated otherwise. A Kaplan–Meier survival curve was used to evaluate the survival of the three clusters up until day 28 and a log-rank test was performed.

Analyses were repeated for the subcohort of patients who had complete data on esophageal manometry measurements.

As a sensitivity analysis, prone position responders were defined as those with an improvement of at least 10% in the main respiratory parameters studied. We also assessed the evolution of main respiratory parameters after returning to the supine position.

As a secondary sensitivity analysis, to account for patients with missing secondary outcome, for each secondary outcome and each patient, a weight was computed as the inverse of the probability of having missing data for the specific outcome and weighted Pearson’s chi-squared tests were computed.

Additionally, a post-hoc power analysis was performed to confirm that the study was adequately powered (80%) to detect a difference of at least 10% in responder rates, if such a difference existed.

This study was approved by the Institutional Review Board of Beth Israel Deaconess Medical Center (2024P000813), and the requirement for informed consent was waived. This study is compliant with the STROBE guidelines for reporting observational studies [31].

Data extraction was performed using Stata (version MP 16.0, StataCorp LLC, College Station, TX, USA) and analyses were performed using R Statistical Software (version 4.2.2, R Foundation for Statistical Computing, Vienna, Austria) with the FactomineR package [32]*.*

## Results

### Study population

Four hundred and sixteen patients fulfilled inclusion criteria and 63 patients were excluded due to missing data on ventilator parameters needed to compute mechanical power (Fig. 1). Baseline characteristics of the 353 patients included in analyses are shown in Table 1. The median age was 62 years (Interquartile IQR: 52–69), patients were mostly male (60%), with a positive RT-PCR COVID-19 test (71%) and a median body mass index (BMI) of 31 (IQR: 26–36) kg/m^2^. Mean tidal volume in supine position was 6.1 (IQR: 5.8–6.6) ml/kg predicted body weight, with a median PEEP of 12 (IQR: 10–14) cmH_2_O. One hundred forty-nine patients died by day 28 (42.2%).

In our cohort, 248 (70.2%) of patients underwent only one session of prone positioning, while 105 (29.8%) received two or more sessions. Median duration of first session of proning was 16 (IQR: 6–22) hours, with a time to proning of 54 (IQR:18–128) hours.

Prone position responders were 41% for respiratory system compliance, 41% for driving pressure, 49% for ventilatory ratio and 42% for mechanical power. Seventy nine percent of patients had an improved PF ratio during prone position.

An esophageal balloon was placed and transpulmonary pressure measurement were available in 173 (49%) patients.

### Factorial analysis of mixed data

In the factorial analysis of mixed data, the most important variables for the first component included ventilatory ratio, plateau pressure, respiratory rate, and for the second component respiratory system compliance, driving pressure, and positive end-expiratory pressure. The number of components to be retained for the factor analysis of mixed data was determined to be 17, based on the optimal number identified through the scree plot (E-Fig. 1).

The results of the visual analysis indicated that the optimal number of clusters was three, as a higher number of clusters was accompanied by only a minimal increase in the explained variance. The percentages of missing values imputed through the factorial analysis of mixed data are shown in E-Fig. 8.

### Hierarchical ascending clustering

Eighty-one patients were assigned to Cluster 1 (22.9%), 181 to Cluster 2 (51.3%), and 91 to Cluster 3 (25.8%) (Table 1).

Regarding the description of the clusters, the most important qualitative variables in characterizing Cluster 1 were the COVID-19 status, with a lower proportion of positive COVID-19 status (46.9% vs. 71.3% in the overall population, p < 0.001) and male gender (72.8% vs. 60.0% in the overall population, p < 0.001). Cluster 2 was defined by a higher proportion of positive COVID-19 patients (84.5% vs 71.3% in overall population, p < 0.001) and Cluster 3 by a higher proportion of patients with restrictive pulmonary disease (24.2% vs 9.9%, p < 0.001).

For continuous variables, Cluster 1 was defined by a higher mean PaO_2_/FiO_2_ ratio (184 mmHg vs. 135 mmHg in the overall population) and a lower PEEP (8 cmH_2_O vs. 12 cmH_2_O in the overall population). Cluster 2 was characterized by a lower driving pressure (12 cmH_2_O vs. 13 cmH_2_O in overall population), higher PEEP (13 cmH_2_O vs. 12 cmH_2_O in the overall population) and a higher respiratory system compliance (36 ml/cmH_2_O vs. 33 ml/cmH_2_O).

**Fig. 2:**
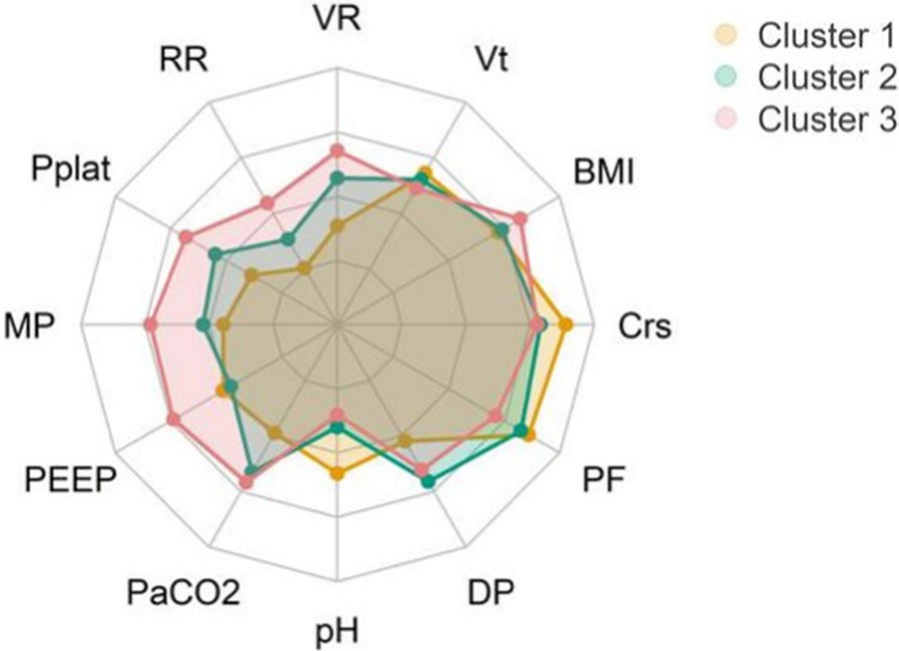
Profiles of different phenotypes according to the most important variables used to determine clusters. Data were normalized to have a mean of 0 and a standard deviation of 1 across all phenotypes. When multiple values were present for a given variable, they were summarized by its median value. Abbreviations: VR ventilatory ratio; Vt tidal volume; BMI body mass index; Crs respiratory system compliance, PF PaO_2_/FiO_2_ ratio; DP driving pressure; pH arterial pH; PaCO_2_ arterial pressure in CO_2_; PEEP positive end-expiratory pressure; MP mechanical power; Pplat plateau pressure; RR respiratory rate

Finally, Cluster 3 was characterized by a lower pH (7.26 vs 7.32), higher PaCO_2_ (61 mmHg vs. 51 mmHg), and higher ventilatory ratio (2.9 vs 2.2) (Fig. 2, E-Table 2–4).

**Table 2:** Clinical outcome and response rate to prone position. Responders are defined by a strictly positive increase or decrease in the parameter of interest

Characteristic	Overall *N* = 353^a^	Cluster 1 *N* = 81^a^	Cluster 2 *N* = 181^a^	Cluster 3 *N* = 91^a^	*p*-value^b^	q-value^c^
Mortality By Day 28	149/353 (42%)	33/81 (41%)	65/181 (36%)	51/91 (56%)	0.006	0.037
Respiratory Compliance Responders	128/313 (41%)	21/54 (39%)	71/175 (41%)	36/84 (43%)	0.9	> 0.9
Driving Pressure Responders	129/313 (41%)	22/54(41%)	70/175 (40%)	37/84 (44%)	0.8	> 0.9
Mechanical Power Responders	130/313 (42%)	21/54 (39%)	74/175 (42%)	35/84 (42%)	> 0.9	> 0.9
PaO_2_/FiO_2_Ratio Responders	234/296 (79%)	32/43 (74%)	139/169 (82%)	63/84 (75%)	0.3	0.9
Ventilatory Ratio Responders	144/295 (49%)	22/41 (52%)	83/169 (49%)	39/84 (46%)	0.8	> 0.9

^a^n (%)

^b^Pearson’s Chi-squared test

^c^False discovery rate correction for multiple testing

Similarly, three clusters were defined for the esophageal pressure subcohort using transpulmonary pressure measurements, with similar covariates differentiating the clusters.

The results for the esophageal pressure subcohort are shown in the supplementary material (E-Table 5–6). Considering the data provided using esophageal pressure, the three clusters shared similar lung and chest wall mechanics characteristics when compared to those of the full population.

### Primary and secondary outcomes

Only 28-day mortality was significantly different across the three clusters (p = 0.037), as shown in Table 2. The highest mortality was in cluster 3 (56%), the lowest mortality was in Cluster 2 (35.9%), and Cluster 1 was intermediate (40.7%). There were no significant differences in the proportions of compliance, driving pressure, PaO_2_/FiO_2_ ratio, ventilatory ratio, or mechanical power responders.

In the esophageal pressure subcohort, Cluster 3 also had a higher mortality rate (p = 0.04), but there were no significant differences in respiratory parameter responders, including transpulmonary pressure and lung compliance.

The trajectories of the primary respiratory parameters were plotted in supine, prone, and return-to-supine positions (E-Fig. 2–6).

For each cluster, there were no significant changes upon prone position for the ventilatory ratio, mechanical power, and driving pressure. Compared to Cluster 1 and 2, Cluster 3 had higher supine values for Cluster 3. This cluster also had a lower supine respiratory system compliance.

There were no significant changes in the esophageal pressure subcohort, with lower supine values for lung compliance and chest wall compliance in Cluster 3. Regarding outcomes, only mortality significantly differed among the three clusters. Cluster 3 had the highest mortality rate.

The Kaplan–Meier survival curves demonstrate distinct differences in survival probabilities over time among the study groups, with Cluster 3 showing significantly lower survival rate compared to Cluster 1 and 2 (E-Fig. 7).

For the definition of prone position responders as an improvement of at least 10% in the respiratory parameters studied, there were also no differences between the three clusters (E-Table 7).

Results were similar when accounting for patients with missing data for each of the secondary outcome (E-Table 8**)**.

The results of the post-hoc power analysis (E-Table 9) confirmed that the study was well-powered, with an 80% likelihood of detecting a minimum 10% difference in responder rates, supporting the robustness of the findings. A sensitivity analysis with the pressure-controlled ventilation equation for mechanical power showed no significant difference between responders for the three clusters (E-Table 11).

## Discussion

In this retrospective observational study, using a machine learning clustering algorithm we identified 3 subphenotypes of patients with ARDS based on respiratory mechanics, oxygenation parameters, and demographic variables in the supine position. The distinct subphenotypes, which are biologically and physiologically coherent and associated with varying mortality rates, displayed no differences in their response rates to proning for the parameters that were evaluated as part of this study. These parameters, which are often considered predictors of a better outcome, might not be related to the observed difference of mortality in our study. It has been reported that the improvement in oxygenation was not associated with a better outcome if the PaO_2_/FiO_2_ ratio was computed over two days after the prone position [33] and also failed to predict improved survival [34]. The benefits of prone positioning through ventilator-induced lung injury (VILI) prevention, lung recruitment, and homogenization of lung aeration [35] might not be captured through punctual bedside measurements, such as in this retrospective study. Transpulmonary pressure measurements did not improve the capacity to discriminate among these cluster subphenotypes of responders but increased the internal validity of the clusters by being reproducible [36]. Adding different sources of data, including longitudinal data or morphological data with lung ultrasound assessment [17], might provide more information and help derive more specific clusters.

Nonetheless, these clusters presented different prognoses and could be clinically relevant for prognostication, particularly Cluster 3. The patients in Cluster 3 presented with a higher disease severity, such as a higher SOFA score and lower PaO_2_/FiO_2_ ratio, but their main characteristic was the occurrence of a pH lower than 7.3 with high levels of PCO_2_ and a median respiratory rate of 30 breaths/min. Increasing the tidal volume in these patients might have been limited by the median plateau pressure of 29.5 cmH_2_O at 6 ml/kg of tidal volume per predicted body weight.

Cluster 3 might represent a subphenotype of patients with relevant pulmonary dead space, which causes hypercapnia and acidosis and could explain, in part, the worse outcome. Prone position may also increase the ventilation perfusion mismatch in persistent ARDS patients [37], which also presents the highest ventilatory ratio, a surrogate for dead space [28]. A high proportion of patients in our cohort with COVID-19 ARDS, which increased dead-space ventilation by provoking alteration of the pulmonary vascular bed, could explain those results [38].

This study has several limitations. First, there was a relatively low number of patients undergoing prone positioning prior to the COVID-19 pandemic. However, change in practice during the COVID-19 pandemic have led to a major increase in the use of the prone position in the world, also at our center. This resulted in a high number of patients being included in this study during the COVID-pandemic [12]. Second, the study was monocentric, and the practice of esophageal pressure measurements and prone position might hinder the generalizability of the clusters. Nonetheless, the use standardized proning protocol enhances the internal validity of our findings and offers a framework that can be replicated in other critical care settings.

Furthermore, regarding the generalizability of our results, the observed 42% mortality rate in our cohort is consistent with international studies, such as the LUNG SAFE [1] study and the ROSE [39] trial, which reported similar mortality rates among ARDS patients.

Third, because all patients underwent prone positioning, no heterogeneity in the strategy effect was studied.

Fourth, patients in cluster 1 and 2 had no significant differences in total respiratory compliance, tidal volume, and driving pressure. Distinction between the two clusters arises not from difference in ventilator settings or ARDS severity but from different risk factors at baseline, with higher Elixhauser score, age, and proportion of male patients in the cluster 1.

Fifth, our analysis was limited to the first proning session to ensure independent samples, a requirement for clustering; while this approach does not capture responses to subsequent sessions, it allows for robust identification of subphenotypes with potential clinical implications. The PROSEVA trial demonstrated that the response to prone positioning is not associated with patient outcomes, and prone positioning should be continued regardless of oxygenation improvement. Similarly, our findings should not suggest discontinuation of prone positioning based solely on these early responses to the first session of prone position.

Sixth, no radiologic or inflammatory biomarkers data was used to build the clusters. While our clustering analysis was based on routinely available clinical variables, future research incorporating anthropometric, biological, morphological, radiologic, and longitudinal data could enhance the precision and applicability of proned position patients subphenotyping.

Seventh, while mortality was chosen as the primary outcome due to its clinical relevance and simplicity, we also analyzed lung-oriented outcomes such as respiratory system compliance and mechanical power to provide a comprehensive understanding of the effects of prone positioning. Notably, the clustering analysis in our study was blinded to outcomes, being constructed solely from baseline variables and respiratory parameters during the supine position, ensuring it was independent of the choice of primary or secondary outcomes.

## Conclusion

In this study, we successfully identified distinct subphenotypes of ARDS patients undergoing prone positioning using unsupervised machine learning based on respiratory mechanics, transpulmonary pressures, and gas exchange parameters. No association was observed between these subphenotypes and mortality benefit or response to prone positioning. These findings underscore the need for continued efforts in phenotyping ARDS through multimodal data to better understand the heterogeneity of this population. Until further evidence is available, prone positioning should remain a cornerstone of treatment for moderate-to-severe ARDS [4], as its broad mortality benefits have been well-established.

## Supplementary Information

Additional file1 (DOCX 812 KB)

## Data Availability

Requests of qualified researchers trained in human subject research and confidentiality to access additional documents and the dataset may be sent to the corresponding author.
