# Supplementary material for: Subphenotyping prone position responders with machine learning
Source: Crit Care. 2025 Mar 14;29:116. doi: 10.1186/s13054-025-05340-8 (PMC11909901; doi:10.1186/s13054-025-05340-8)
Supplement: Supplementary file 1 — Additional file1 (DOCX 812 KB) [file 13054_2025_5340_MOESM1_ESM.docx]

**ELECTRONIC SUPPLEMENTARY MATERIAL**

**Subphenotyping prone position responders with machine learning**

Maxime Fosset^1,2,3,4#^, Dario von Wedel^1,2,5#^, Simone Redaelli^1,2,6,7#^, Daniel Talmor^1,2^, Nicolas Molinari^4^, Julie Josse^4^, Elias N Baedorf-Kassis^8^, Maximilian S Schaefer^1,2,9^, Boris Jung^1,2,3,8^

^1^Department of Anesthesia, Critical Care and Pain Medicine, Beth Israel Deaconess Medical Center, Harvard Medical School, Boston, MA, USA

^2^Center for Anesthesia Research Excellence (CARE), Beth Israel Deaconess Medical Center, Harvard Medical School, Boston, MA, USA

^3^Medical Intensive Care Unit and PhyMedExp, Montpellier University Hospital, Montpellier, France

^4^Desbrest Institute of Epidemiology and Public Health, University of Montpellier, INRIA, Montpellier, France

^5^Institute of Medical Informatics, Charité - Universitätsmedizin Berlin, Berlin, Germany

^6^School of Medicine and Surgery, University of Milano-Bicocca, Milan, Italy

^7^Department of Anesthesiology, Perioperative and Pain Medicine, Lahey Hospital and Medical Center, Burlington, MA, USA

^8^Department of Pulmonary, Critical Care & Sleep Medicine, Beth Israel Deaconess Medical Center, Harvard Medical School, Boston, MA, USA

^9^Department of Anesthesiology, Duesseldorf University Hospital, Duesseldorf, Germany

^#^Contributed equally to this work

**E-Figure 1: Scree plot of the factorial analysis of mixed data and description of the clustering algorithm**

**E-Figure 2: Evolution of Respiratory System Compliance for the three clusters across stages of prone position**

**E-Figure 3: Evolution of Driving Pressure for the three clusters across stages of prone position**

**E-Figure 4: Evolution of Mechanical Power for the three clusters across stages of prone position**

**E-Figure 5: Evolution of** PaO_2_/FIO_2_**ratio for the three clusters across stages of prone position**

**E-Figure 6: Evolution of Ventilatory Ratio for the three clusters across stages of prone position**

**E-Figure 7: Kaplan-Meier curve of the survival probabilities for the three clusters**

**E-Figure 8: Missing values percentages for variables imputed through factorial analysis of mixed data**

**E-Table 1: Extract from the BIDMC protocol for Prone Position in the ICU**

**E-Table 2: Description of the cluster 1 with their variables**

**E-Table 3: Description of the cluster 2 with their variables**

**E-Table 4: Description of the cluster 3 with their variables**

**E-Table 5: Characteristics of the population and of the three clusters for the sub population with available esophageal pressure measurements**

**E-Table 6: Response rate for mortality, respiratory mechanics and oxygenation parameters by cluster and overall for the sub population with available esophageal pressure measurements**

**E-Table 7: Response rate for respiratory mechanics and oxygenation parameters by cluster and overall for the full population with a definition of improvement of at least 10 %**

**E-Table 8: Response rate to proning position adjusted for missing secondary outcomes**

**E-Table 9: Post-hoc power analysis for response rate**

**E-Table 10: Obstructive and restrictive lung diseases definition**

**E-Table 11: Mechanical power responders rate with Pressure-Controlled Ventilation equation**

**E-Figure 1: Scree plot of the factorial analysis of mixed data and description of the clustering algorithm**

**
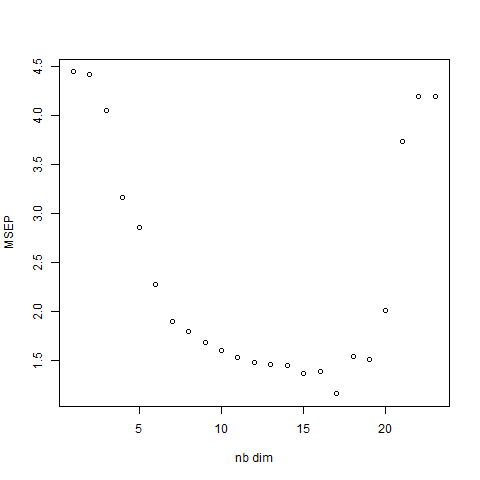
**

**Scree plot for the number of dimensions:**

A number of dimensions of 17 was chosen as the one with the lowest mean square error, meaning that most of the variance in the response variable is explained by a number of 17 components

MSEP = Mean Square Error of Prediction

Nb dim = Number of dimensions

**Description of the clustering algorithm:**

We used factorial analysis of mixed data, an extension of principal component analysis suited to both qualitative and mixed data. This approach allows to summarize the information by reducing the dimensionality of the data without losing important information. A new set of variables is created, so-called principal components (scores), which capture the maximum variance in the data. This scree plot with cross-validation was used to determine the optimal number of components. As the number of dimensions increases, the model’s predictive accuracy is improving, with the lower point at 17 dimensions. Increasing dimensions over this number might decrease the performance of the algorithm.

This denoising method serves as a preprocessing tool for the unsupervised clustering technique that we chose, that is, hierarchical ascending clustering.

The hierarchical clustering method was then used to define three clusters and was built by computing the Euclidean distance between individuals and using the Ward criterion to minimize the variance at each step of the construction of the algorithm and homogenize the clusters.

The optimal number of clusters was determined visually and through the between-cluster variance increase, when the number of clusters was increased by one.

The clusters are described in two ways.

First, for continuous variables, each variable was described by its mean, and a v-test was performed, with the p-value corresponding to the test of the following hypothesis:” The mean of the category is equal to the overall mean.”

This test compares the mean of a variable within a cluster to the global mean, accounting for variance and sample size. It does not require the assumption of normal data distribution, leveraging the central limit theorem for its approximation to normality in larger samples​.

For categorical variables, a χ2 test was performed between the categorical variables and the cluster variable, and a p-value less than 0.05 showing that the categorical variable was linked to the cluster variable.

Further, the link between the cluster and quantitative variables was also computed as the correlation ratio.

**E-Figure 2: Evolution of Respiratory System Compliance for the three clusters across stages of prone position**


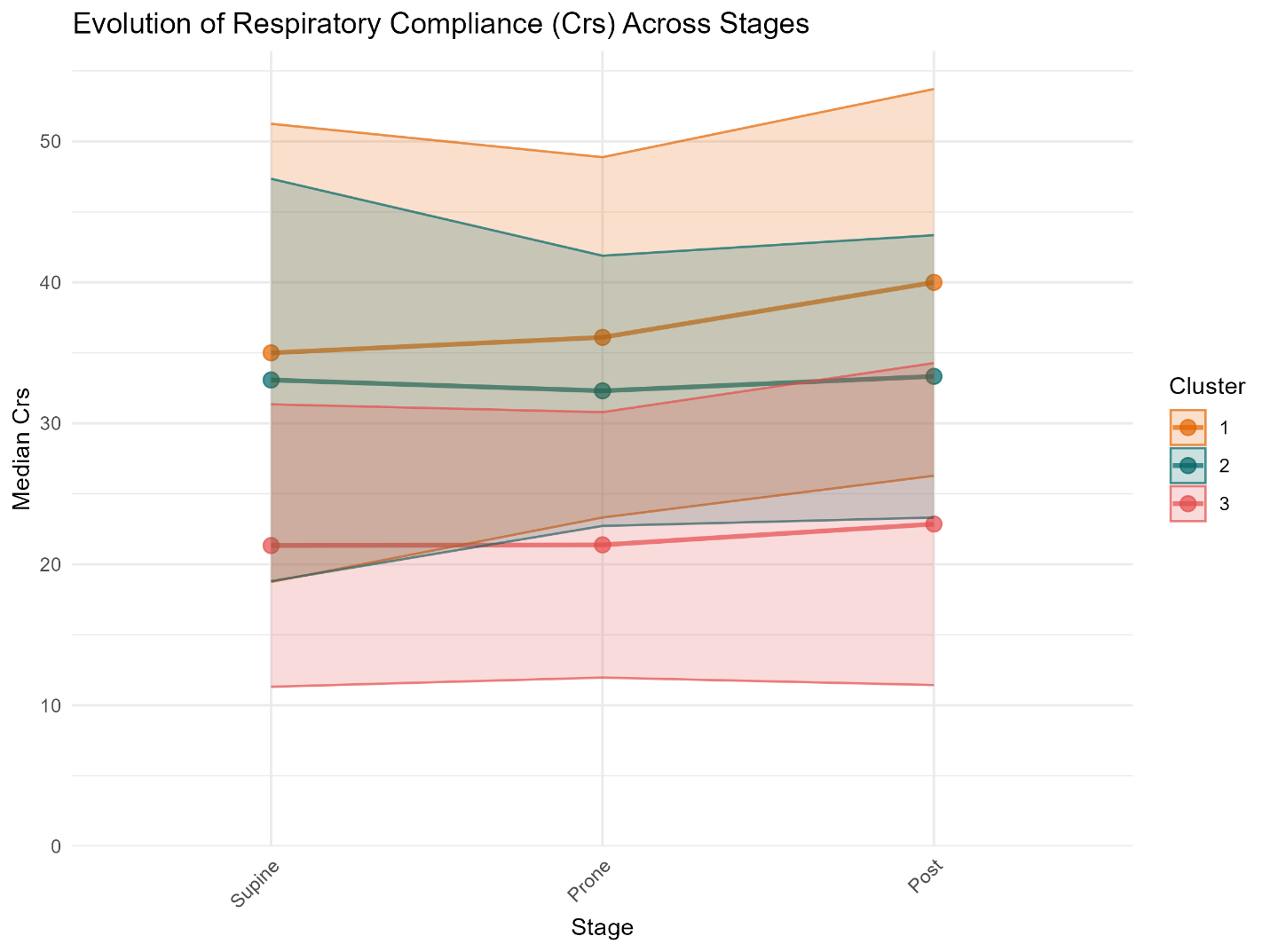


Temporal trends across three phases: baseline (Supine), intervention (Prone), and follow-up (Post). Each colored line represents the mean values across the study cohort for the respective clusters. The shaded regions around each line illustrate the 95% confidence intervals (CIs).

**E-Figure 3: Evolution of Driving Pressure for the three clusters across stages of prone position**


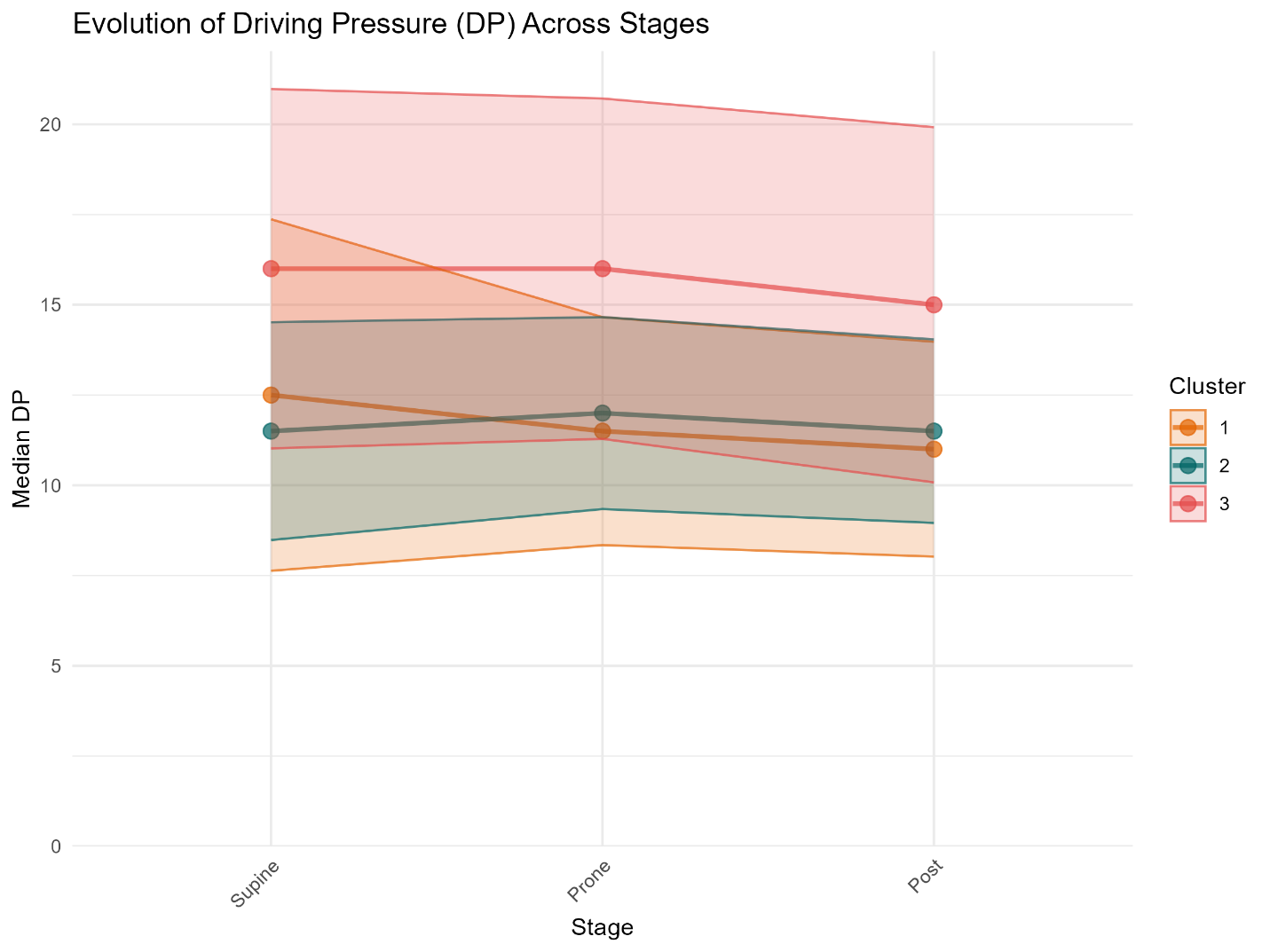


Temporal trends across three phases: baseline (Supine), intervention (Prone), and follow-up (Post). Each colored line represents the mean values across the study cohort for the respective clusters. The shaded regions around each line illustrate the 95% confidence intervals (CIs).

**E-Figure 4: Evolution of Mechanical Power for the three clusters across stages of prone position**


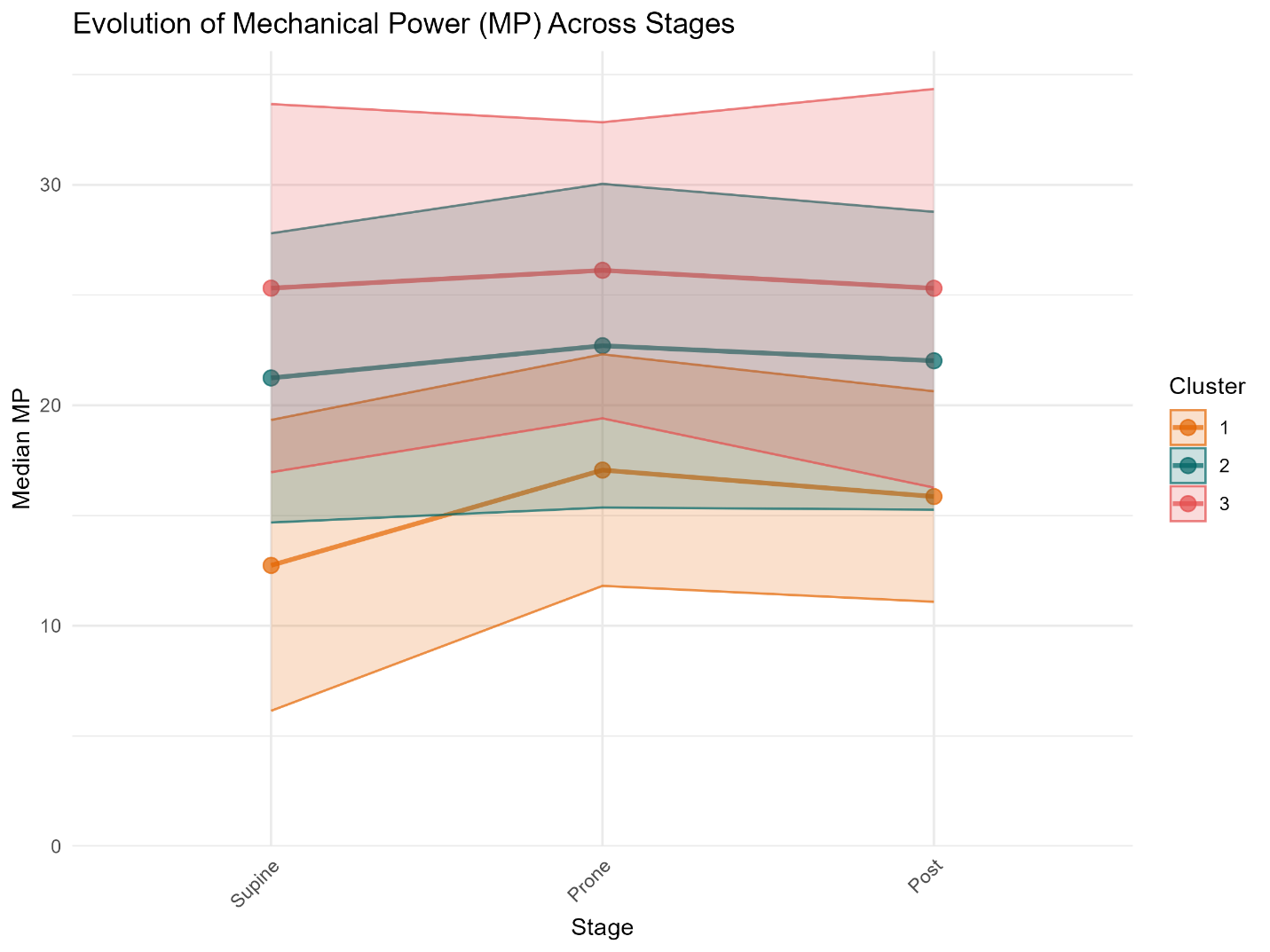


Temporal trends across three phases: baseline (Supine), intervention (Prone), and follow-up (Post). Each colored line represents the mean values across the study cohort for the respective clusters. The shaded regions around each line illustrate the 95% confidence intervals (CIs).

**E-Figure 5: Evolution of** PaO_2_/FIO_2_**ratio for the three clusters across stages of prone position**

**
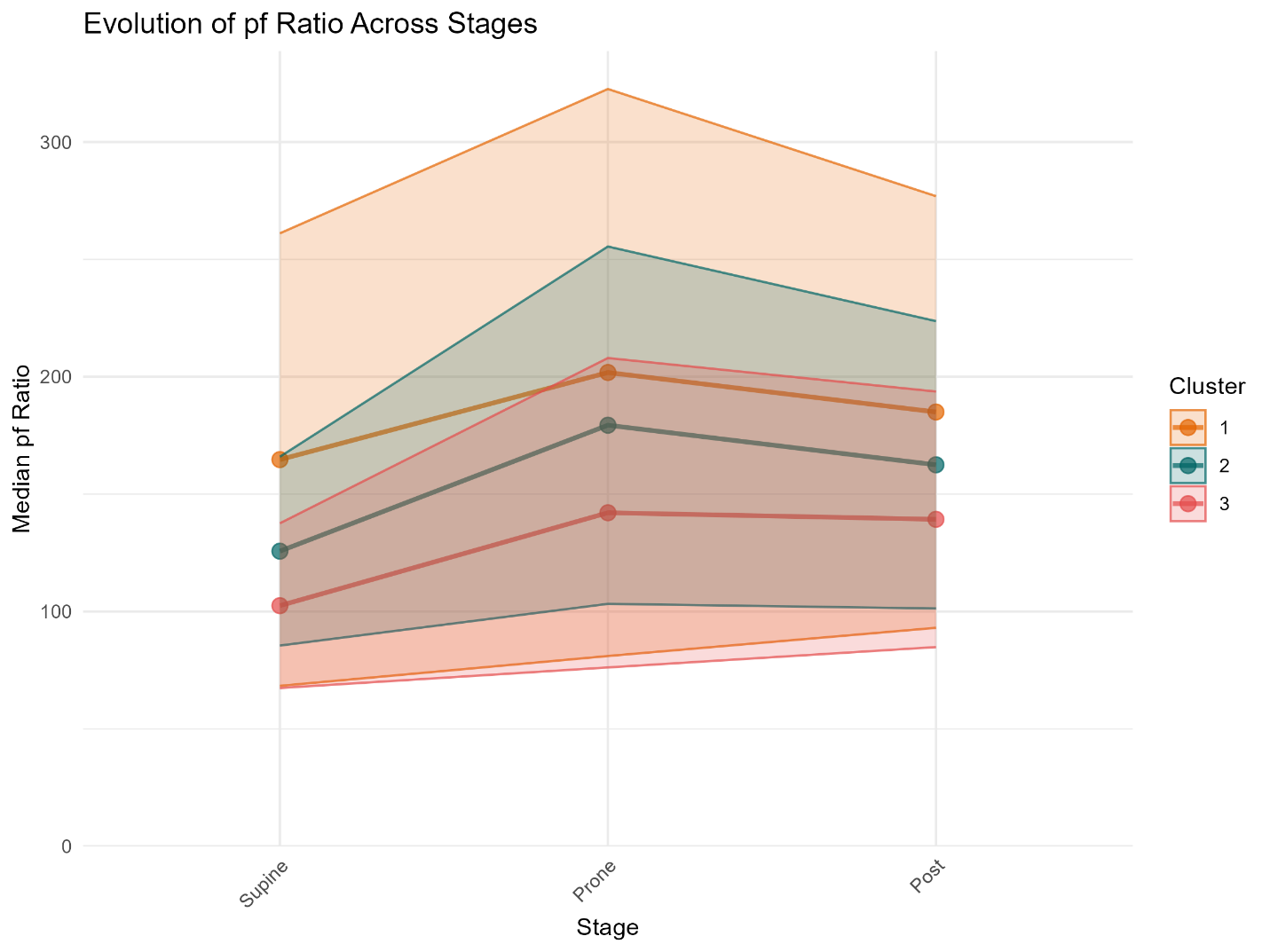
**

Temporal trends across three phases: baseline (Supine), intervention (Prone), and follow-up (Post). Each colored line represents the mean values across the study cohort for the respective clusters. The shaded regions around each line illustrate the 95% confidence intervals (CIs).

**E-Figure 6: Evolution of Ventilatory Ratio for the three clusters across stages of prone position**


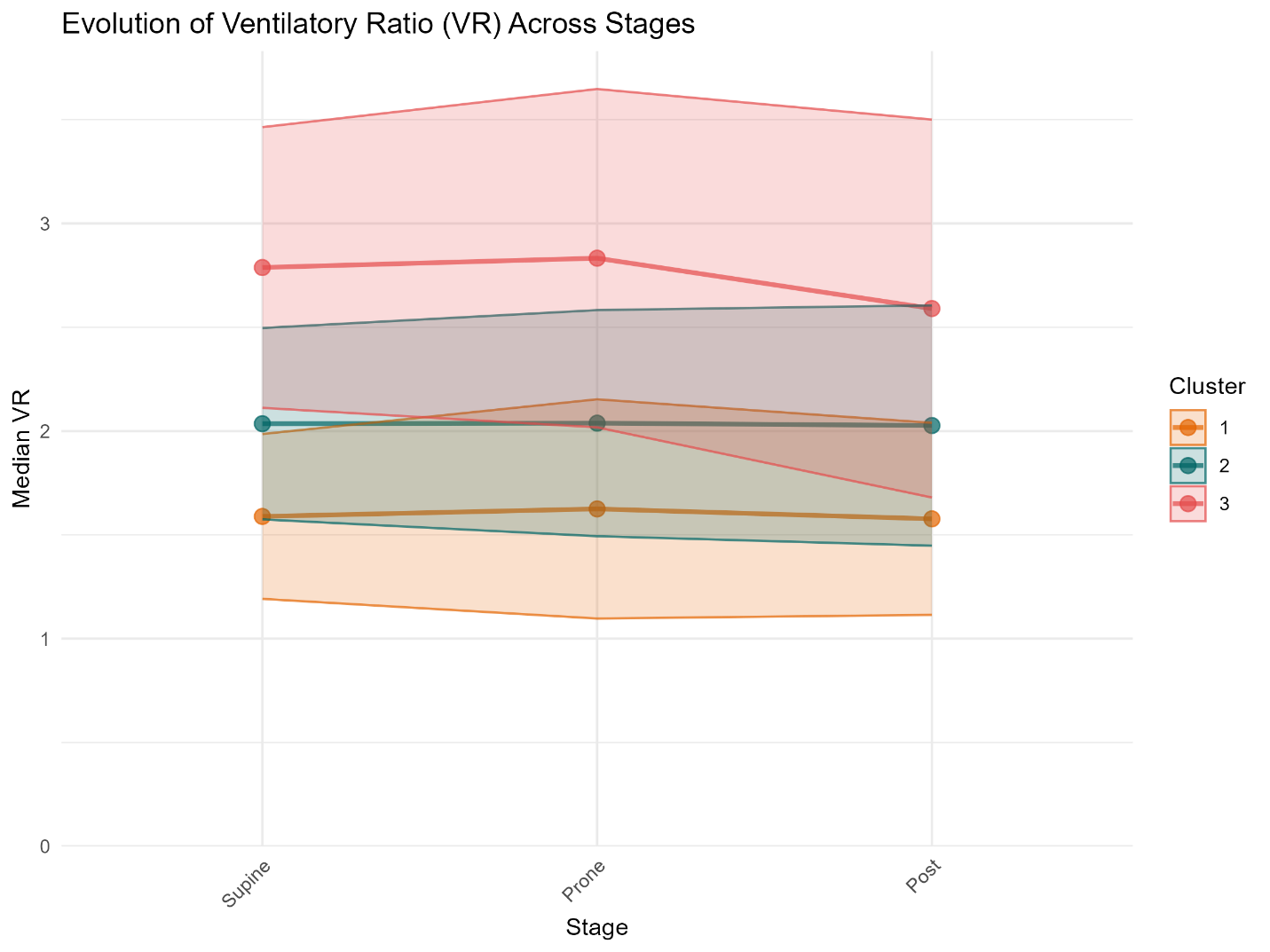


Temporal trends across three phases: baseline (Supine), intervention (Prone), and follow-up (Post). Each colored line represents the mean values across the study cohort for the respective clusters. The shaded regions around each line illustrate the 95% confidence intervals (CIs).

**E-Figure 7: Kaplan-Meier curve of the survival probabilities for the three clusters**

**
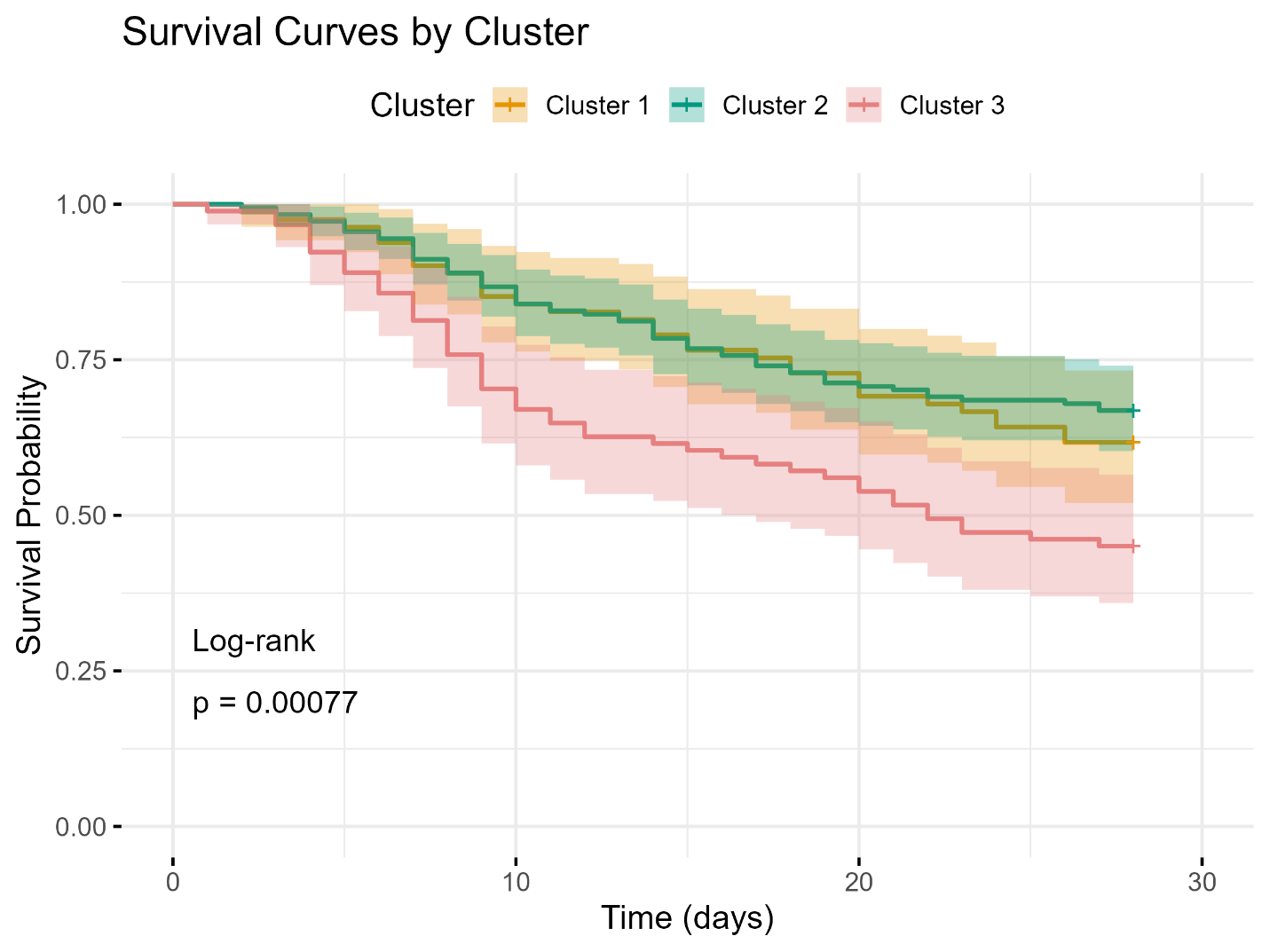
**

P-value shown is for the log rank test.

**E-Figure 8: Missing values percentages for variables imputed through factorial analysis of mixed data**


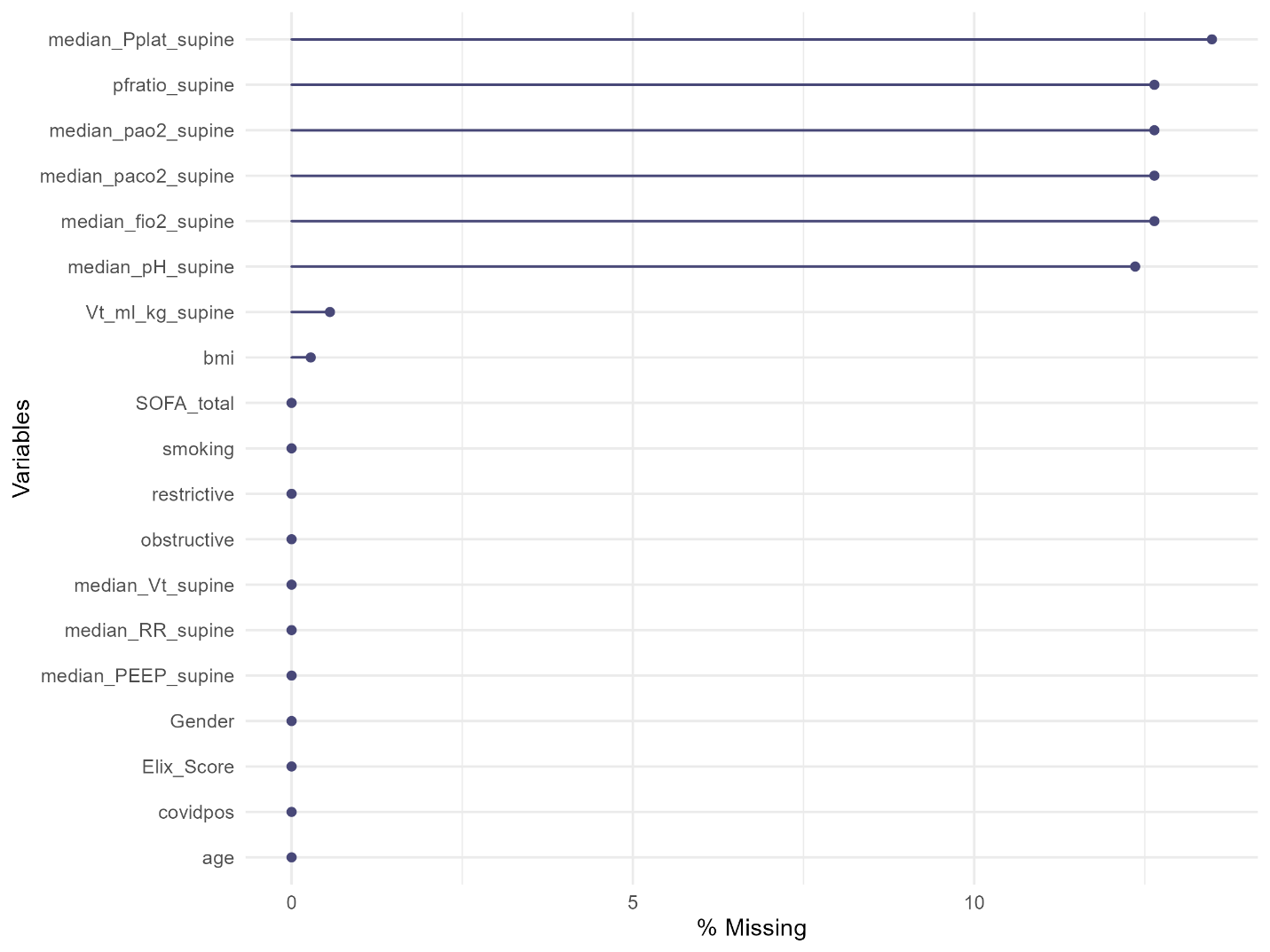


**E-Table 1: Extract from the BIDMC protocol for Prone Position in the ICU**

**Policy Statement**

• Physician or advance practice provider must order prone position

**Indications**

• Acute Respiratory Distress Syndrome (ARDS) with PaO2:FiO2 (P/F) ratio

<150mmHg and FiO2 >60% with PEEP ≥ 5

• Non-intubated patient with escalating oxygen requirements who is able to

self-prone

• Severe posterior wound management

• Sleep promotion/comfort for patients who sleep in the prone position at

baseline and are able to self-prone

**Contraindications**

• Unstable spine fractures

• Uncontrolled ICP

Precautions needed with the following patient populations:

• Open chest or unstable chest wall

• Extracorporeal membrane oxygenation (ECMO)

• Unstable pelvis, skull, or facial fracture(s)

• Open abdomen or distended abdomen and/or ischemic bowel

• Elevated ICP

• Cervical/skeletal traction in place

• Hemodynamic instability

• Weight > 160kg

• Pregnancy

• Bifurcated endotracheal tube (ETT) or tracheostomy

• Arm/shoulder injury

**Complications**

• Hemodynamic instability

• Catheter, tube, or drain dislodgement, displacement, or obstruction

• Pressure injury

• Peripheral nerve injury

• Corneal ulceration

• Facial/orbital edema

**Recommendations**

• Initiation of prone position for intubated patients with ARDS:

o Best within 24-36 hours of severe ARDS diagnosis, but up to eight

days after severe ARDS develops

• Duration of prone position for intubated patients with ARDS:

o It is recommended that a patient be proned for at least 16 consecutive

hours in a 24-hour period

• Discontinuation of prone position for intubated patients with ARDS:

o Patient improvement:

▪ When, four hours after being supinated, the patient's PaO_2_/FIO_2_ ratio is

≥ 150mmHg at FiO2 ≤60% and PEEP ≤10cm of water

o Patient does not tolerate treatment

▪ Decrease in PaO_2_/FIO_2_ ratio of >20% relative to the ratio in the supine

position (from x2 prone sessions prior)

▪ Unresolved hemodynamic instability

▪ Interruption of life sustaining therapy

**E-Table 2: Description of the cluster 1 with their variables**

Only the variables with significant link are showed, in descending order; variables are sorted according to the correlation ratio (between the quantitative variable and the cluster variable)

| Variable | Mean in category | Standard deviation in category | Overall mean | Overall standard deviation | V.test value | p-value |
| --- | --- | --- | --- | --- | --- | --- |
| PaO2/FIO2ratio (mmHg) | 184.2 | 95.8 | 135.3 | 63.3 | 7.9 | <0.001 |
| Tidal Volume (ml) | 433.7 | 98.6 | 388.7 | 79.0 | 5.8 | <0.001 |
| pH | 7.36 | 0.06 | 7.32 | 0.08 | 4.8 | <0.001 |
| Elixhauser Score | 29.2 | 12.5 | 23.8 | 11.6 | 4.7 | <0.001 |
| Tidal Volume per kg (ml/kg) | 6.6 | 1.3 | 6.2 | 0.9 | 4.5 | <0.001 |
| PaO_2_ (mmHg) | 103.6 | 37.3 | 92.9 | 26.8 | 4.1 | <0.001 |
| Compliance (ml/ cmH_2_O) | 37.3 | 16.1 | 32.9 | 14.9 | 3.0 | 0.003 |
| BMI (kg/m²) | 28.2 | 7.2 | 32.6 | 8.5 | -5.3 | <0.001 |
| PaCO_2_ (mmHg) | 45.4 | 8.2 | 51.1 | 11.0 | -5.3 | <0.001 |
| FiO_2_ (%) | 62.5 | 17.5 | 74.5 | 17.7 | -6.9 | <0.001 |
| Ventilation Ratio | 1.6 | 0.4 | 2.2 | 0.7 | -8.3 | <0.001 |
| Plateau Pressure (cmH_2_O) | 21.1 | 4.5 | 25.2 | 4.8 | -8.8 | <0.001 |
| Respiratory Rate (breaths/min) | 21.1 | 4.4 | 26.0 | 5.1 | -9.9 | <0.001 |
| Mechanical Power (J/min) | 12.6 | 6.6 | 20.8 | 8.5 | -10.0 | <0.001 |
| PEEP (cmH_2_O) | 7.9 | 2.9 | 11.8 | 3.9 | -10.3 | <0.001 |

**E-Table 3: Description of the cluster 2 with their variables**

Only the variables with significant link are showed, in descending order; variables are sorted according to the correlation ratio (between the quantitative variable and the cluster variable)

| Variable | Mean in category | Standard deviation in category | Overall mean | Overall standard deviation | V.test value | p-value |
| --- | --- | --- | --- | --- | --- | --- |
| PEEP (cmH_2_O) | 13.2 | 2.8 | 11.8 | 3.9 | 7.0 | <0.001 |
| Compliance (ml/ cmH_2_O) | 35.8 | 14.2 | 32.9 | 14.9 | 3.8 | <0.001 |
| pH | 7.34 | 0.07 | 7.32 | 0.08 | 3.7 | <0.001 |
| Mechanical Power (J/min) | 22.1 | 6.5 | 20.8 | 8.5 | 2.8 | 0.005 |
| BMI (kg/m²) | 33.5 | 7.7 | 32.6 | 8.5 | 2.1 | 0.040 |
| PaO_2_ (mmHg) | 89.4 | 22.0 | 92.9 | 26.8 | -2.5 | 0.013 |
| PaO2/FIO2ratio (mmHg) | 126.4 | 40.1 | 135.3 | 63.3 | -2.7 | 0.007 |
| Ventilation Ratio | 2.1 | 0.5 | 2.2 | 0.7 | -2.8 | 0.006 |
| PaCO2 (mmHg) | 48.6 | 8.2 | 51.1 | 11.0 | -4.3 | <0.001 |
| Elixhauser Score | 21.0 | 10.9 | 23.8 | 11.6 | -4.7 | <0.001 |
| Driving Pressure (cmH_2_O) | 11.7 | 3.0 | 13.4 | 4.6 | -7.2 | <0.001 |

**E-Table 4: Description of the cluster 3 with their variables**

Only the variables with significant link are showed, in descending order; variables are sorted according to the correlation ratio (between the quantitative variable and the cluster variable)

| Variable | Mean in category | Standard deviation in category | Overall mean | Overall standard deviation | V.test value | p-value |
| --- | --- | --- | --- | --- | --- | --- |
| Ventilation Ratio | 2.9 | 0.7 | 2.2 | 0.7 | 11.1 | <0.001 |
| PaCO_2_ (mmHg) | 61.1 | 11.9 | 51.1 | 11.0 | 10.0 | <0.001 |
| Plateau Pressure (cmH_2_O) | 29.5 | 4.1 | 25.2 | 4.8 | 9.9 | <0.001 |
| Respiratory Rate (breaths/min) | 30.1 | 3.8 | 26.0 | 5.1 | 9.0 | <0.001 |
| Driving Pressure (cmH_2_O) | 17.0 | 5.0 | 13.4 | 4.6 | 8.7 | <0.001 |
| FiO_2_ (%) | 86.6 | 14.1 | 74.5 | 17.7 | 7.6 | <0.001 |
| Mechanical Power (J/min) | 25.7 | 8.3 | 20.8 | 8.5 | 6.4 | <0.001 |
| BMI (kg/m²) | 34.6 | 9.6 | 32.6 | 8.5 | 2.7 | 0.007 |
| SOFA Score | 10.0 | 5.2 | 9.1 | 4.6 | 2.2 | 0.025 |
| PEEP (cmH_2_O) | 12.5 | 4.2 | 11.8 | 3.9 | 2.0 | 0.049 |
| Tidal Volume per kg (ml/kg) | 5.9 | 0.8 | 6.2 | 0.9 | -3.9 | <0.001 |
| Tidal Volume (ml) | 356.6 | 67.6 | 388.7 | 79.0 | -4.5 | <0.001 |
| PaO2/FIO2ratio (mmHg) | 109.6 | 34.9 | 135.3 | 63.3 | -4.5 | <0.001 |
| Compliance (ml/ cmH_2_O) | 23.2 | 10.0 | 32.9 | 14.9 | -7.2 | <0.001 |
| pH | 7.26 | 0.08 | 7.32 | 0.08 | -8.8 | <0.001 |

**E-Table 5: Characteristics of the population and of the three clusters for the sub population with available esophageal pressure measurements**

| **Characteristic** | **Overall**  N = 173*^1^* | **Cluster 1**  N = 82*^1^* | **Cluster 2**  N = 46*^1^* | **Cluster 3**  N = 45*^1^* |
| --- | --- | --- | --- | --- |
| Age (years) | 58 (49, 66) | 61 (50, 69) | 52 (43, 61) | 60 (53, 67) |
| Gender (female) | 71 (41%) | 31 (38%) | 16 (35%) | 24 (53%) |
| COVID-19 Positive | 140 (81%) | 71 (87%) | 37 (80%) | 32 (71%) |
| Body Mass Index (BMI, kg/m²) | 33 (29, 38) | 31 (28, 35) | 38 (33, 47) | 31 (28, 38) |
| Elixhauser Comorbidity Score | 18 (11, 27) | 19 (9, 28) | 16 (10, 23) | 19 (14, 27) |
| Obstructive Lung Disease | 49 (28%) | 22 (27%) | 9 (20%) | 18 (40%) |
| Restrictive Lung Disease | 19 (11%) | 3 (3.7%) | 1 (2.2%) | 15 (33%) |
| Smoking Status | 65 (38%) | 32 (39%) | 16 (35%) | 17 (38%) |
| Total SOFA Score | 10 (7, 13) | 10 (6, 12) | 11.0 (10.0, 15) | 10 (4, 13) |
| Respiratory Rate (breaths/min) | 28 (24, 30) | 25 (23, 28) | 28 (25, 30) | 30 (26, 33) |
| Median PEEP (cmH_2_O) | 12 (10, 15) | 12 (10, 14) | 16 (15, 20) | 12 (10, 12) |
| Median Tidal Volume (mL) | 370 (320, 420) | 380 (340, 420) | 400 (370, 465) | 320 (300, 360) |
| Median Plateau Pressure (cmH_2_O) | 26 (24, 29) | 24 (22, 25) | 29 (27, 31) | 29 (27, 32) |
| Mechanical Power (J/min) | 22 (18, 27) | 20 (17, 22) | 30 (25, 34) | 23 (19, 26) |
| Median PaO_2_ (mmHg) | 86 (76, 100) | 86 (76, 100) | 91 (79, 102) | 82 (72, 95) |
| Median FiO_2_ (%) | 80 (60, 100) | 70 (60, 85) | 85 (70, 100) | 90 (80, 100) |
| PaO2/FIO2Ratio | 113 (91, 139) | 131 (100, 156) | 113 (96, 133) | 95 (83, 107) |
| Median PaCO_2_ (mmHg) | 51 (44, 59) | 47 (42, 52) | 54 (47, 61) | 57 (50, 65) |
| Median pH | 7.32 (7.26, 7.37) | 7.35 (7.32, 7.4) | 7.29 (7.22, 7.34) | 7.3 (7.22, 7.32) |
| Driving Pressure (cmH_2_O) | 13 (11, 15) | 11 (10, 13) | 12 (11, 13) | 18 (15, 20) |
| Respiratory System Compliance (mL/cmH_2_O) | 30 (23, 38) | 33 (29, 41) | 34 (30, 39) | 20 (16, 23) |
| Tidal Volume per kg (mL/kg) | 6.1 (5.7, 6.4) | 6.1 (5.9, 6.4) | 6.3 (6, 6.9) | 5.7 (5.1, 6.1) |
| Ventilatory Ratio | 2.2 (1.8, 2.7) | 1.8 (1.6, 2.2) | 2.5 (2.2, 3.2) | 2.5 (2.1, 2.8) |
| Transpulmonary expiratory pressure (mmHg) | 1 (-0.5, 2) | 1.5 (0, 2) | 0.7 (-1, 2) | 1 (-0.9, 2) |
| Transpulmonary inspiratory pressure (mmHg) | 11 (8.5, 13) | 9.7 (7.8, 12.1) | 9.9 (7.7, 11.9) | 14.2 (12.6, 16.5) |
| Transpulmonary driving pressure (mmHg) | 10 (7.5, 12.5) | 8.8 (7, 10.3) | 9 (7.5, 10.5) | 13.6 (12.3, 16.2) |
| Lung compliance (mL/cmH_2_O) | 38 (29, 49) | 45 (36, 57) | 45 (35, 49) | 24 (21, 29) |
| End Inspiratory Esophageal Pressure (mmHg) | 15.2 (12.5, 18) | 14 (11.5, 15.8) | 19 (17.2, 21.9) | 14.5 (11.7, 17) |
| End Expiratory Esophageal Pressure (mmHg) | 12.1 (9.7, 14.6) | 10.6 (8.6, 13) | 16 (14.1, 18.5) | 10.7 (8.7, 12.5) |
| Chest Wall Compliance (mL/cmH_2_O) | 108 (68, 168) | 111 (74, 167) | 148 (101, 224) | 89 (58, 124) |
| Predicted Body Weight (kg) | 60 (52, 66) | 60 (55, 66) | 61 (55, 66) | 57 (50, 64) |
| *^1^*Median (IQR); n (%) | | | | |

When several measurements were available, the median was computed for each patient. Respiratory parameters were recorded in the supine position.

**E-Table 6: Response rate for mortality, respiratory mechanics and oxygenation parameters by cluster and overall for the sub population with available esophageal pressure measurements**

Responders are defined by a strictly positive increase or decrease in the parameter of interest

| **Characteristic** | **Overall**  N = 173*^1^* | **Cluster 1**  N = 82*^1^* | **Cluster 2**  N = 46*^1^* | **Cluster 3**  N = 45*^1^* | **p-value***^2^* | **q-value***^3^* |
| --- | --- | --- | --- | --- | --- | --- |
| Mortality By Day 28 | 72/173 (42%) | 27/82 (33%) | 17/46 (37%) | 28/45 (62%) | 0.004 | 0.040 |
| Respiratory Compliance Responders | 72/170 (42%) | 35/81 (43%) | 18/45 (40%) | 19/44 (43%) | >0.9 | >0.9 |
| Driving Pressure Responders | 73/170 (43%) | 33/81  (41%) | 20/45 (44%) | 20/44 (45%) | 0.9 | >0.9 |
| Mechanical Power Responders | 74/170 (44%) | 34/81 (42%) | 22/45 (49%) | 18/44 (41%) | 0.7 | >0.9 |
| PaO2/FIO2Ratio Responders | 132/169 (78%) | 64/80 (80%) | 36/46 (78%) | 32/43 (74%) | 0.8 | >0.9 |
| Ventilatory Ratio Responders | 92/169 (54%) | 43/80 (54%) | 31/46 (67%) | 18/43 (42%) | 0.053 | 0.2 |
| Transpulmonary Driving Pressure Responders | 66/118 (56%) | 30/50 (59%) | 19/33 (58%) | 17/34 (50%) | 0.7 | >0.9 |
| Lung Compliance Responders | 63/118 (53%) | 29/50 (57%) | 18/33 (55%) | 16/34 (47%) | 0.7 | >0.9 |
| Chest Wall Compliance Responders | 35/118 (30%) | 14/50 (27%) | 8/33 (24%) | 13/34 (38%) | 0.4 | >0.9 |
| *^1^*n (%) | | | | | | |
| *^2^*Pearson's Chi-squared test | | | | | | |
| *^3^*False discovery rate correction for multiple testing | | | | | | |

**E-Table 7: Response rate for respiratory mechanics and oxygenation parameters by cluster and overall for the full population with a definition of improvement of at least 10%**

Responders are defined by an increase or decrease strictly greater than 10% in the parameter of interest

| **Characteristic** | **Overall**, N = 353*^1^* | **Cluster 1**, N = 81*^1^* | **Cluster 2**, N = 181*^1^* | **Cluster 3**, N = 91*^1^* | **p-value***^2^* | **q-value***^3^* |
| --- | --- | --- | --- | --- | --- | --- |
| Respiratory Compliance Responders | 69/313 (22%) | 11/54 (20%) | 39/175 (22%) | 19/84 (23%) | >0.9 | >0.9 |
| Driving Pressure Responders | 72/313 (23%) | 15/54 (28%) | 38/175 (22%) | 19/84 (23%) | 0.6 | >0.9 |
| Mechanical Power Responders | 62/313 (20%) | 10/54 (19%) | 34/175 (19%) | 18/84 (21%) | 0.9 | >0.9 |
| PaO2/FIO2Ratio Responders | 217/296 (73%) | 26/43 (60%) | 133/169 (79%) | 58/84 (69%) | 0.032 | 0.2 |
| Ventilatory Ratio Responders | 76/295 (26%) | 9/41 (21%) | 41/169 (24%) | 26/84 (31%) | 0.4 | >0.9 |
| *^1^*n (%) | | | | | | |
| *^2^*Pearson's Chi-squared test | | | | | | |
| *^3^*False discovery rate correction for multiple testing | | | | | | |

**E-Table 8: Response rate to proning position adjusted for missing secondary outcomes**

Responders are defined by a strictly positive increase or decrease in the parameter of interest.

To account for patients with missing secondary outcome, for each secondary outcome and each patient, a weight was computed as the inverse of the probability of having missing data for the specific outcome. Probabilities of missing data were computed through multivariable logistic regression including the baseline covariates. Then a weighted Pearson’s chi-square test with Rao and Scott adjustment was performed to assess the difference between the proportions of responders.

| **Characteristic** | **Overall**  N = 353*^1^* | **Cluster 1**  N = 81*^1^* | **Cluster 2**  N = 181*^1^* | **Cluster 3**  N = 91*^1^* | **p-value***^2^* |
| --- | --- | --- | --- | --- | --- |
| Respiratory Compliance Responders | 128/313 (41%) | 21/54 (39%) | 71/175 (41%) | 36/84 (43%) | 0.7 |
| Driving Pressure Responders | 129/313 (41%) | 22/54  (41%) | 70/175 (40%) | 37/84(44%) | 0.3 |
| Mechanical Power Responders | 130/313  (42%) | 21/54 (39%) | 74/175 (42%) | 35/84 (42%) | 0.5 |
| PaO2/FIO2Ratio Responders | 234/296 (79%) | 32/43  (74%) | 139/169 (82%) | 63/84 (75%) | 0.2 |
| Ventilatory Ratio Responders | 144/295  (49%) | 22/41 (52%) | 83/169 (49%) | 39/84 (46%) | 0.8 |

| *^1^*n (%) |
| --- |
| *^2^*Pearson's Chi-squared test with Rao and Scott adjustment |
|  |

**E-Table 9: Post-hoc power analysis for response rate**

| **Statistic** | **Observed proportions** | **Expected proportions** | **Cohen’s w** | **Degrees of freedom** | **Significance level (α)** | **Power (1-β)** | **Total Sample Size)** | **Sample size per cluster** |
| --- | --- | --- | --- | --- | --- | --- | --- | --- |
| Value | 60%,50%,40% | 50%,50%,50% | 0.2 | 2 | 0.05 | 0.8 | 241 | 81 |

To evaluate the association between cluster membership and responder status, we calculated Cohen’s w to quantify the effect size based on observed responder proportions across the three clusters, with a decreasing number of responders for the cluster with the highest mortality (Cluster 1: 60%, Cluster 2: 50%, Cluster 3: 40%).

The expected proportions under the null hypothesis of no association were evenly distributed across clusters $(E=50\%)$.

Cohen’s w was computed as:

$$w=\sqrt{\sum\frac{\left( O_{i}-E_{i} \right)^{2}}{E_{i}}}$$

A subsequent power analysis, using a chi-square test with df=2, a significance level of α=0.05, and a target power of 1−β=0.8, estimated that a total sample size of 241 participants would be required to achieve sufficient power. Assuming equal distribution across clusters, this corresponds to a minimum of 81 participants per cluster.

**E-Table 10: Obstructive and restrictive lung diseases definition**

| **ICD-10 Code** | **Diagnostic Description** |
| --- | --- |
| **Obstructive Lung Diseases** |  |
| J41.1 | Mucopurulent chronic bronchitis |
| J41.8 | Mixed simple and mucopurulent chronic bronchitis |
| J42 | Unspecified chronic bronchitis |
| J43.1 | Panlobular emphysema |
| J43.2 | Centrilobular emphysema |
| J43.8 | Other emphysema |
| J43.9 | Emphysema, unspecified |
| J44.0 | Chronic obstructive pulmonary disease with (acute) lower respiratory infection |
| J44.1 | Chronic obstructive pulmonary disease with (acute) exacerbation |
| J44.9 | Chronic obstructive pulmonary disease, unspecified |
| J45 | Asthma |
| J47.0 | Bronchiectasis with acute lower respiratory infection |
| J47.1 | Bronchiectasis with (acute) exacerbation |
| J47.9 | Bronchiectasis, uncomplicated |
| **Restrictive Lung Diseases** |  |
| D86.0 | Sarcoidosis of lung |
| M30.1 | Polyarteritis nodosa |
| M34.81 | Systemic sclerosis with lung involvement |
| M33.91 | Dermatopolymyositis, unspecified with respiratory involvement |
| M05.10 | Rheumatoid lung disease with rheumatoid arthritis of unspecified site |
| M32.13 | Lung involvement in systemic lupus erythematosus |
| J60 | Coalworker's pneumoconiosis |
| J61 | Pneumoconiosis due to asbestos and other mineral fibers |
| J62 | Pneumoconiosis due to dust containing silica |
| J63 | Pneumoconiosis due to other inorganic dusts |
| J64 | Unspecified pneumoconiosis |
| J65 | Pneumoconiosis associated with tuberculosis |
| J84.01 | Idiopathic pulmonary fibrosis |
| J84.02 | Idiopathic pulmonary alveolar proteinosis |
| J84.03 | Idiopathic non-specific interstitial pneumonitis |
| J84.09 | Other interstitial pulmonary diseases with fibrosis in diseases classified elsewhere |
| J84.841 | Lymphangioleiomyomatosis |
| J84.83 | Surfactant mutations of the lung |
| J84.89 | Other specified interstitial pulmonary diseases |

Obstructive lung diseases were identified by the presence of ICD-10 codes associated with airflow obstruction (e.g., COPD, asthma, and bronchiectasis). Restrictive lung diseases were identified based on codes for interstitial lung diseases, pneumoconiosis, and systemic diseases with pulmonary involvement. These definitions were based on diagnostic coding in electronic health records (EHRs) used in this study.

**E-Table 11: Mechanical power responders rate with Pressure-Controlled Ventilation equation**

As a sensitivity analysis, we used the Pressure-Controlled Ventilation (PCV) equation of mechanical power described by Becher et al [1] for the overall population and the clusters.

$$\mathrm{MP}_{\mathrm{PCV}}=0.098\cdot\mathrm{RR}\cdot V_{T}\cdot\left( \Delta P_{\mathrm{insp}}+\mathrm{PEEP} \right)$$

| **Characteristic** | **Overall**  N = 353*^1^* | **Cluster 1**  N = 81*^1^* | **Cluster 2**  N = 181*^1^* | **Cluster 3**  N = 91*^1^* | **p-value***^2^* |
| --- | --- | --- | --- | --- | --- |
| Mechanical Power (PCV Equation) | 26.7 (21.6 – 33.1) | 17.7 (13.1-23.5) | 26.8 (23.3-31.8) | 33.9 (27.9 – 40.2) |  |
| Mechanical Power (PCV Equation) Responders | 146/353  (41%) | 31/81 (38%) | 74/181 (41%) | 41/91 (45%) | 0.66 |

| *^1^*n (%) or median (IQR)  *^2^* Chi-square test |
| --- |
|  |

REFERENCES

1. Becher T, van der Staay M, Schädler D, Frerichs I, Weiler N. Calculation of mechanical power for pressure-controlled ventilation. Intensive Care Med. 2019;45:1321‑3.
